# Transcriptome Analysis of Secondary Metabolism Pathway, Transcription Factors, and Transporters in Response to Methyl Jasmonate in *Lycoris aurea*

**DOI:** 10.3389/fpls.2016.01971

**Published:** 2017-01-05

**Authors:** Rong Wang, Sheng Xu, Ning Wang, Bing Xia, Yumei Jiang, Ren Wang

**Affiliations:** ^1^Institute of Botany, Jiangsu Province and Chinese Academy of SciencesNanjing, China; ^2^The Jiangsu Provincial Platform for Conservation and Utilization of Agricultural GermplasmNanjing, China; ^3^Key Laboratory of Biology and Genetic Improvement of Soybean, National Center for Soybean Improvement, Ministry of Agriculture, Nanjing Agricultural UniversityNanjing, China

**Keywords:** Amaryllidaceae alkaloids, *Lycoris aurea*, methyl jasmonate, transcriptome sequencing, secondary metabolite

## Abstract

*Lycoris aurea*, a medicinal species of the Amaryllidaceae family, is used in the practice of traditional Chinese medicine (TCM) because of its broad pharmacological activities of Amaryllidaceae alkaloids. Despite the officinal and economic importance of *Lycoris* species, the secondary mechanism for this species is relatively deficient. In this study, we attempted to characterize the transcriptome profiling of *L. aurea* seedlings with the methyl jasmonate (MeJA) treatment to uncover the molecular mechanisms regulating plant secondary metabolite pathway. By using short reads sequencing technology (Illumina), two sequencing cDNA libraries prepared from control (Con) and 100 μM MeJA-treated (MJ100) samples were sequenced. A total of 26,809,842 and 25,874,478 clean reads in the Con and MJ100 libraries, respectively, were obtained and assembled into 59,643 unigenes. Among them, 41,585 (69.72%) unigenes were annotated by basic local alignment search tool similarity searches against public sequence databases. These included 55 Gene Ontology (GO) terms, 128 Kyoto Encyclopedia of Genes and Genomes (KEGG) pathways, and 25 Clusters of Orthologous Groups (COG) families. Additionally, 4,175 differentially expressed genes (DEGs; false discovery rate ≤ 0.001 and |log_2_ Ratio| ≥ 1) with 2,291 up-regulated and 1,884 down-regulated, were found to be affected significantly under MeJA treatment. Subsequently, the DEGs encoding key enzymes involving in the secondary metabolite biosynthetic pathways, transcription factors, and transporter proteins were also analyzed and summarized. Meanwhile, we confirmed the altered expression levels of the unigenes that encode transporters and transcription factors using quantitative real-time PCR (qRT-PCR). With this transcriptome sequencing, future genetic and genomics studies related to the molecular mechanisms associated with the chemical composition of *L. aurea* may be improved. Additionally, the genes involved in the enrichment of secondary metabolite biosynthesis-related pathways could enhance the potential applications of *L. aurea*.

## Introduction

Plants produce the crucial chemicals for growth and development by their own, including the primary and secondary metabolites. The primary metabolites include carbohydrates, acids, amino acids, fat and so on. The secondary metabolites, also named as natural products or phytochemicals, are specific to some taxonomic groups, playing pivotal roles in interactions between the plant and environment, helping plants to defense against pathogens or herbivores (Dixon, 2001; Kennedy and Wightman, 2011; Kliebenstein and Osbourn, 2012). As the sources of drugs, insecticides, and flavor, most of the plant secondary metabolites are very important for human's daily life (Goossens et al., 2003; Hussain et al., 2012; Yang et al., 2012).

*Lycoris aurea*, belonging to Amaryllidaceae family, is a medicinally and ornamentally important species. It is rich of the secondary metabolites such as Amaryllidaceae-type alkaloids, and is widely used for traditional Chinese medicine (TCM). The Amaryllidaceae-type alkaloids which are isolated from *Lycoris* genus have been reported to exhibit immunostimulatory, anti-malarial, tumor, and viral activities (Jin, 2009). For example, as one of the typical Amaryllidaceae alkaloids, galanthamine is a kind of reversible inhibitor of cholinesterase to increase acetylcholine sensitivity, and it has also been clinically used in the treatment of Alzheimer's disease (Harvey, 1995; Bores et al., 1996). Despite of the officinal, economic and cultural importance of *Lycoris* species, the secondary mechanism for this species are relatively limited.

The experimental approach based on sequencing the functional genomics was reported to facilitate gene discovery in plant secondary metabolism (Dixon, 2001; Goossens et al., 2003). Besides, RNA-sequencing (RNA-Seq) technology was used to obtain full-scale transcriptomic information from different plant species such as tea plant, *Polygala tenuifolia, Chlorophytum borivilianum*, and *Atractylodes lancea*, and provide a better insight into transcriptional and post-transcriptional regulation of the essential genes in the secondary metabolite biosynthetic pathways (Kalra et al., 2013; Li et al., 2015; Devi et al., 2016). Regarding to Amaryllidaceae-type alkaloids biosynthesis pathway, Kilgore et al. (2014) defined a 4′-*O*-methyltransferase which is involved in the biosynthesis of galanthamine by sequencing transcriptome of *Narcissus* sp. *aff. Pseudonarcissus*. Previously, the *de novo* transcriptome was sequenced to produce the EST (comprehensive expressed sequence tag) dataset for *Lycoris aurea*, which provides one perspective of the regulatory and synthesized molecular mechanisms of Amaryllidaceae-type alkaloids (Wang et al., 2013). On the other hand, the inspection of the comparative transcriptome files between two databases provides another method to investigate the secondary metabolites biosynthesis, or/and find the objective genes involved in *Salvia miltiorrhiza, Catharanthus roseus*, sweet cherry, *Atractylodes lancea*, and so on (Guo et al., 2013; Yu and Luca, 2013; Wei et al., 2015; Huang et al., 2016).

A great diversity in the types of microbial, physical, or chemical factors, known as elicitors, could influence the levels of secondary metabolites in plants (Zhao et al., 2005; Vasconsuelo and Boland, 2007; Jimenez-Garcia et al., 2013; Verma and Shukla, 2015). For example, the elicitors such as nutrient supply, temperature, metal ions, light conditions and atmospheric CO_2_ concentrations affect the production of secondary metabolites in plants (Nascimento and Fett-Neto, 2010; Gandhi et al., 2015). The elicitors derived from bacteria, fungi, viral pathogens and even plants also contribute to the variability in plant secondary metabolism (Berenbaum, 1995; Verma and Shukla, 2015). Previously, we showed that exogenous methyl jasmonate (MeJA) application accelerated the Amaryllidaceae alkaloids accumulation in *Lycoris chinensis* seedlings (Mu et al., 2009). In this study, by using elicitor MeJA treatment, the global expression patterns of genes involved in metabolism, particularly secondary metabolism, transcription factors, and transporter proteins were identified. Therefore, this transcriptome sequencing may help improve future genetics and genomics studies on molecular mechanisms associated with the secondary metabolites of *L. aurea*.

## Materials and methods

### Plant growth and treatment

*L. aurea* seeds were collected from Institute of Botany, Jiangsu Province and Chinese Academy of Sciences, Nanjing, China. The seeds were surface sterilized with 75% alcohol (v/v), and germinated on half-strength Murashige and Skoog (MS) medium (pH 5.8) in the dark at room temperature for 10 days. Afterwards, the seedlings were transferred into plastic pots containing a mixture of soil and vermiculite (3:1, v/v) and cultured in a growth chamber under 14 h light (25°C)/10 h dark (22°C). After 12 months growth, the seedlings were treated with 100 μmol L^−1^ methyl jasmonate (MJ100) for 6 h. MeJA was dissolved with 1% DMSO (v/v) to prepare the stock solution. Seedlings grown in MeJA-free solution (1% DMSO) were used as control (Con). The seedlings were harvested and immediately frozen in liquid nitrogen and stored at −80°C.

### RNA isolation, cDNA library construction and illumina sequencing

Total RNA of the samples were extracted using RNAiso Plus reagent (Takara Bio, Dalian, China) following the manufacturer's instruction. RNA samples were examined with a spectrophotometer (Thermo Fisher Scientific, Inc. Waltham, MA, USA) and electrophoresed on a 1% agarose gel. The construction of the cDNA libraries and the RNA-Seq assay were performed by the OE Biotech Company (Shanghai, China).

Poly (A) mRNA was enriched referring to the previous method (Yu et al., 2016) by using NEBNext® Poly(A) mRNA Magnetic Isolation Module (New England Biolabs, Ipswich, MA, USA), and fragmented to short pieces. These short fragments were as applied as the templates for cDNA. The cDNAs were then subjected to end-repair using T_4_ DNA polymerase and phosphorylation using Klenow DNA polymerase. Then, a base “A” was added to the 3′ ends of the repaired cDNA fragments. All of the short fragments were linked to sequencing adapter. The resulting fragments were selected as the PCR templates after electrophoresis. Finally, the four libraries were sequenced using Illumina HiSeq™ 2,000.

### Transcriptome assembly and functional annotation

The raw data of the RNA sequencing were purified by trimming adapters, removing reads containing poly-N, and rejecting the low-quality data (quality value ≤ 10 or unknown nucleotides larger than 5%) to get the clean reads. Meanwhile, the proportion of nucleotides with quality values greater than 20 (Q20) and GC content of the clean data were calculated. Then, all of the clean reads were assembled by using Trinity program (Grabherr et al., 2011). Firstly, for each library, the certain short reads with overlap regions were assembled into longer contiguous sequences (contigs). Then, based on the paired-end information, the distance of different contigs was recognized by mapping the clean reads, to obtain the sequence of the transcripts. Finally, performing the sequence of potential transcript to TGI Clustering tool, the unigenes were obtained (Pertea et al., 2003). For gene functional annotation, all of the assembled unigenes were aligned to the public databases, including National Center for Biotechnology Information (NCBI) non-redundant protein (Nr) and nucleotide (Nt) database, the Swiss-Prot protein database, Gene Ontology (GO, http://wego.genomics.org.cn/cgi-bin/wego/index.pl) database, Clusters of Orthologous Groups (COGs) database, and the Kyoto Encyclopedia of Genes and Genomes (KEGG, http://www.genome.jp/kegg/kegg2.html) database, with a cut-off of *E* ≤ 10^−5^.

### Differentially expressed genes (DEGs) analysis

The expression level of unigene was calculated following fragments per kilobase of exon per million fragments mapped reads (FPKM) method (Mortazavi et al., 2008). To identify DEGs between two groups, the ratio of the FPKM values (using 0.001 instead of 0 if the FPKM was 0) were taken as the fold-changes in the expression of each gene. In order to compute the significance of the difference in transcript abundance, the false discovery rate (FDR) control method was used to identify the threshold of the *P*-value in multiple tests (Reiner et al., 2003). In this result, only fold change with|log2 (MJ100_FPKM/Con_FPKM)|≥ 1, and an FDR ≤ 0.001 were taken as the threshold for significantly differential expression. The log_2_-transformed FPKM value for DEGs was applied to generate heat map by MeV 4.7 (Howe et al., 2011). Meanwhile, the DEGs were annotated with GO and KEGG databases.

### Validation of DEGs with quantitative real-time PCR (qRT-PCR)

qRT-PCR was used to confirm the expression of MeJA-responsive genes in *L. aurea*. Total RNA was extracted from tissue sample using the method described above. The first-strand cDNA was synthesized using a PrimeScript™ II First Strand cDNA synthesis kit (Takara Bio, Dalian, China) according to the manufacturer's protocol. The primers were designed using the software Primer Premier 5.0, and listed in Table S1. The quantified expression levels of the tested genes were normalized against the housekeeping genes *Cyclophilin 1* (*CYP1*) (Ma et al., 2016). qRT-PCR was performed using SYBR Premix Ex Taq™ II kit (Takara) and run on qTOWER2.2 Real-Time PCR System (Analytik Jena AG, Jena, Germany). Conditions for quantitative analysis were as follows: 94°C for 2 min; 35 cycles of 94°C for 15 s, 60°C for 20 s, 72°C for 10 min. Data for each sample were calculated using 2^−Δ*ΔCT*^ method (Livak and Schmittgen, 2001).

## Results

### Transcriptome sequencing profile of *L. aura* under MeJA treatment

**Figure 1:**
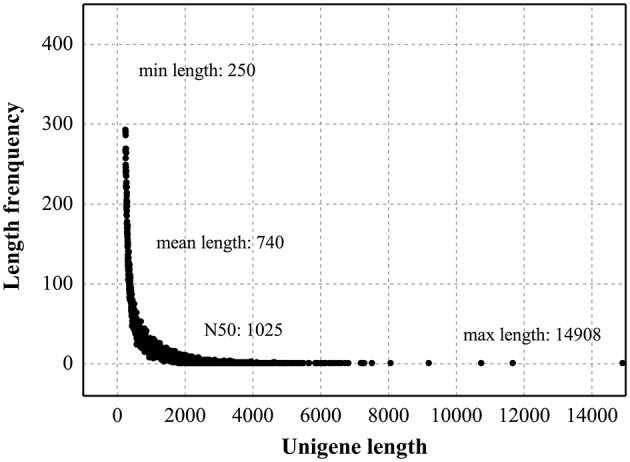
**Length distribution of assembled unigenes**.

To develop a comprehensive overview of the *L. aurea* transcriptome under MeJA treatment, two Solexa/Illumina libraries Con and MJ100, were designed for RNA-Seq. These two libraries (Con and MJ100) produced 4.82 Gbyte and 4.65 Gbyte of clean data, respectively. In addition, paired-end reads of each library are with GC percentage and Q20 percentage of 97.52 and 45.24%, 97.42 and 44.83%, respectively. Subsequently, short reads with average lengths of 244 and 273 bp of the two libraries were collected into 181,881 and 156,621 contigs, respectively (Table 1). Taking the distance of paired-end reads into account, these contigs were assembled into non-redundant unigenes (Table S2). In total, 59,643 unigenes in the range of 250–14,908 bp (with an average length of 740 bp) were obtained (Figure 1).

**Table 1:** **Summary of Illumina HiSeq™ 2,000 assembly and analysis of ***L. aurea*** transcriptome sequences**.

**Samples**	**Raw reads**	**Clean reads**	**Clean bases**	**Q20 percentage (%)**	**GC percentage (%)**	**Average length (bp)**	**Total contigs**
Con	27,257,506	26,809,842	4.82G	97.52	45.24	244	181,881
MJ100	26,191,328	25,874,478	4.65G	97.42	44.83	273	156,621

### Sequence annotation and classification

**Figure 2:**
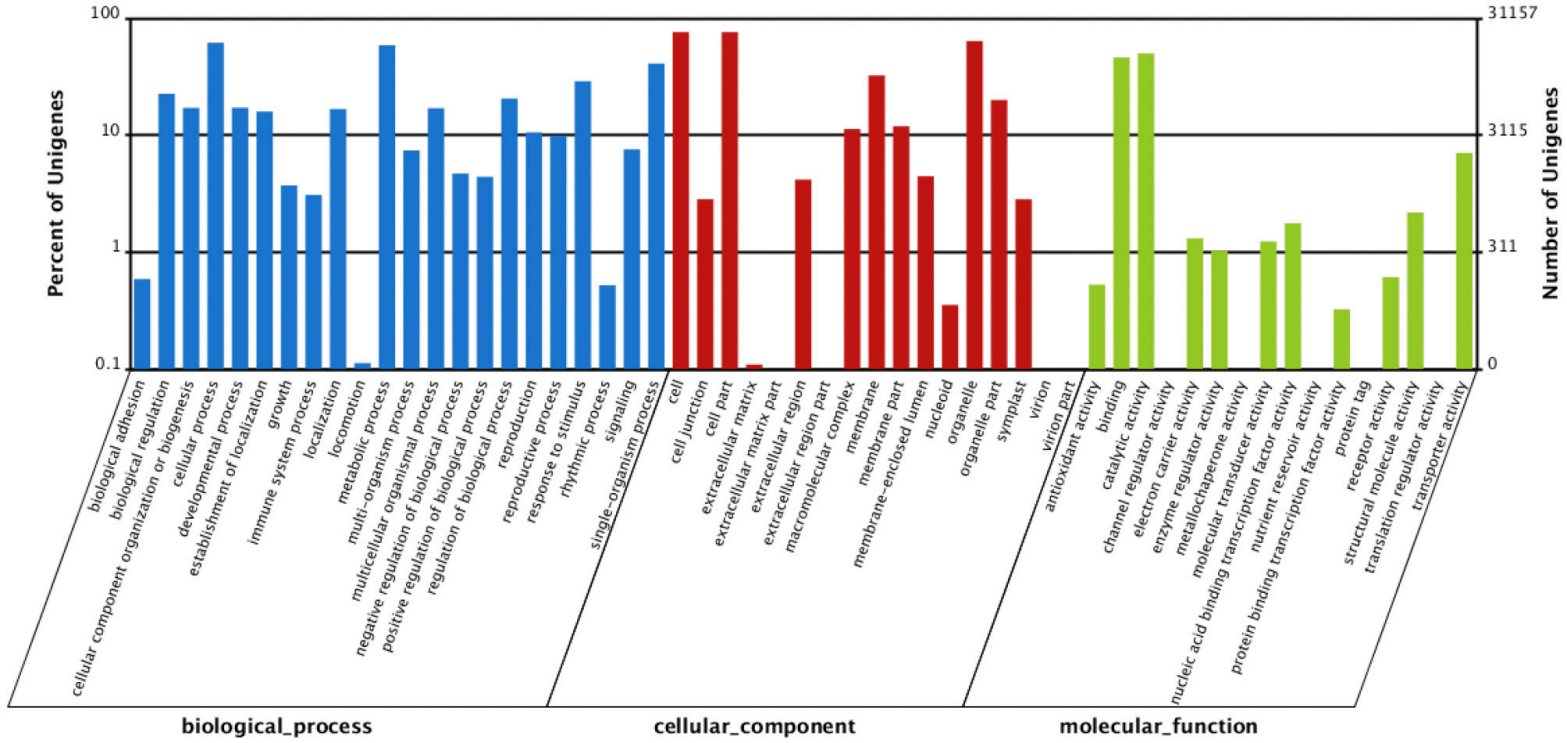
**Gene ontology (GO) categorization of assembled unigenes**. The unigenes were categorized based on gene ontology annotation, and the proportion of each category is displayed based on the ontologies biological process, cellular component, and molecular function.

All of the unigenes were annotated by BLAST search in the public databases (Table 2). The results revealed that 40,636 unigenes (68.13%) had significantly matched in the Nr database, 30,216 (50.66%) in the Nt database, and 26,345 (44.17%) in the Swiss-Prot database. Taken the entire public databases together, there were 41,585 unigenes (69.72%) successfully annotated (Table 2). For GO analysis, there were 31,157 unigenes divided into three ontologies, the percentage and summary number of unigenes was annotated in each GO term shown in Figure 2 and Table S3. Based on the similarity to sequences with known functions, 115,216 sequences were assigned to the biological process (BP) category, 95,500 to the cellular component (CC) category, 34,965 to the molecular function (MF) category. Because several unigenes were assigned to more than one category, the total assigned number was bigger than the total number of the unigenes. In MF category, genes assigned to “catalytic activity” (15,547) and “binding” (14,379) constituted the largest proportion, accounting for 85.59% of the total. Within the CC category, “cell” (23,719), “cell part” (23,717) and “organelle” (19,872) were highly represented. Moreover, “cellular process” (19,233) and “metabolic processes” (18,289) were the main groups under BP category (Figure 2; Table S3).

**Table 2:** **Functional annotation of non-redundant unigenes against the public databases**.

**Annotation database**	**Number of unigenes**	**Percentage (%)**	**300 ≤ length < 1000 nt**	**length ≥ 1000 nt**
Annotated in NR	40,636	68.13	23,381	13,428
Annotated in NT	30,216	50.66	15,969	12,129
Annotated in COG	15,370	25.77	7,034	7,737
Annotated in GO	31,157	52.24	17,468	11,133
Annotated in KEGG	24,651	41.33	12,977	9,851
Annotated in Swiss-Prot	26,345	44.17	14,216	9,994
Annotated in at least one database	41,585	69.72	24,004	13,449
Total unigenes	59,643	100	34,768	13,754

**Figure 3:**
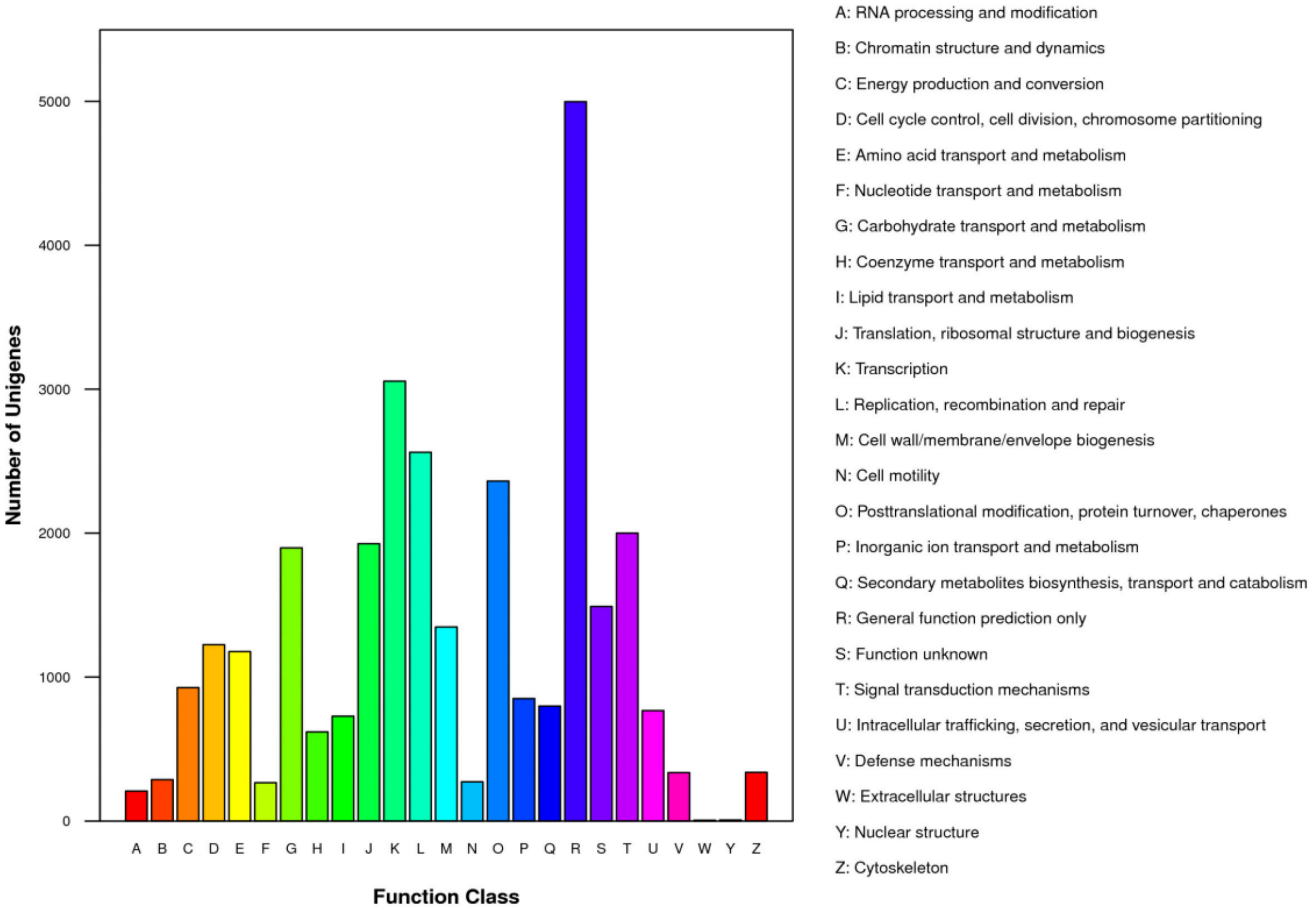
**Clusters of orthologous groups (COG) classifications of putative proteins**. All putative proteins were aligned to the COG database and can be classified functionally into at least 25 molecular families.

**Figure 4:**
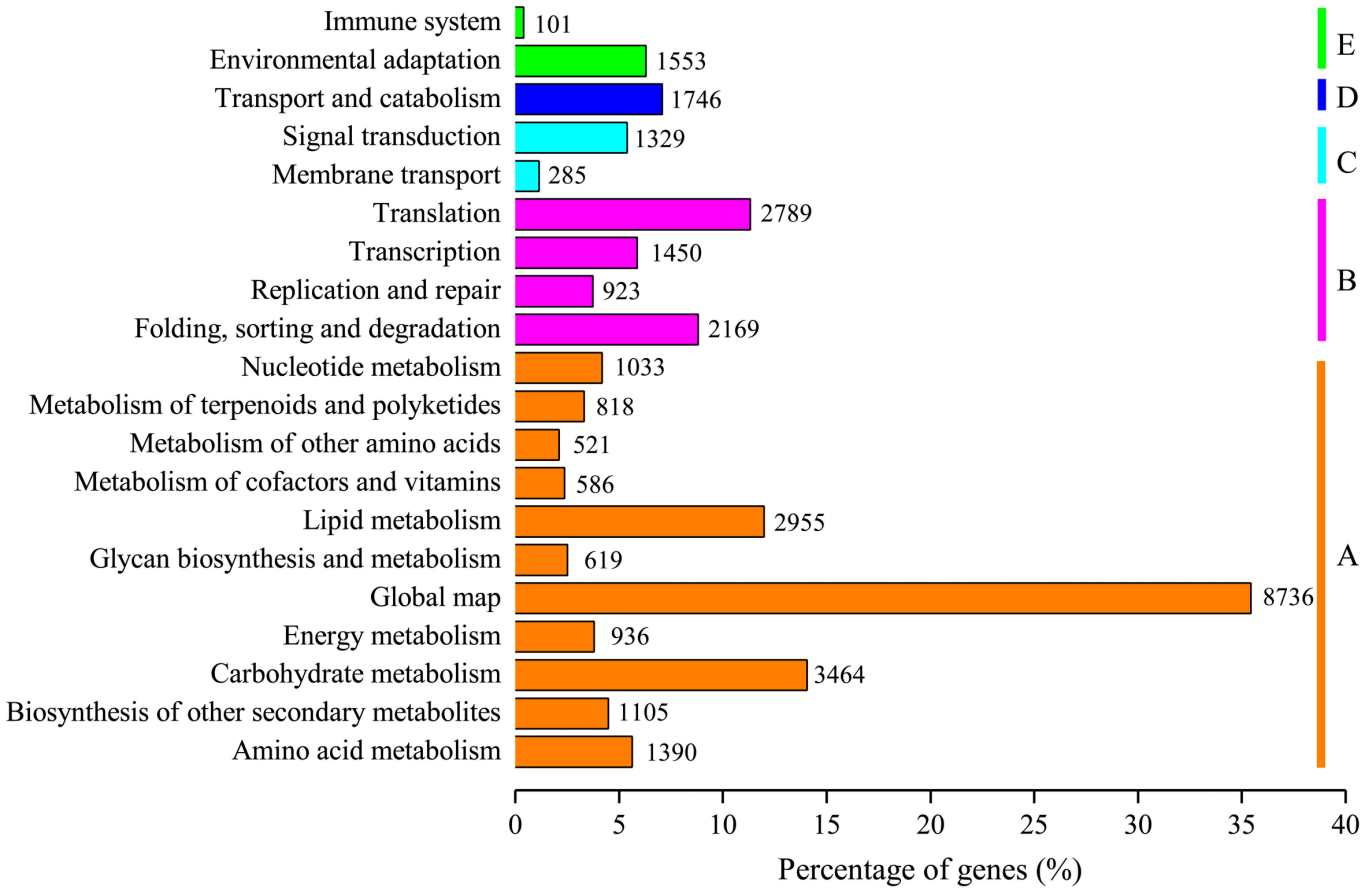
**KEGG metabolism pathway categories of assembled unigenes**. Main functional categories are the Metabolism **(A)**, Genetic Information Processing **(B)**, Environmental Information Processing **(C)**, Cellular Processes **(D)**, and Organismal Systems **(E)**. Bars represent the numbers of *L. aurea* assignments of unigenes with BLASTX matches to each KEGG term.

For functional prediction and classification, all unigenes were subjected to BLAST search against the COG database. There were 15,370 unigenes assigned to COG functional classification and divided into 25 specific categories based on the homology (Table 2; Figure 3). The “General functional prediction only” (4,997) was the largest category, followed by “Transcription” (3,056), “Replication, recombination and repair” (2,562), “Post-translational modification, protein turnover, chaperones” (2,362) and “Signal transduction mechanisms” (2,000). “Nuclear structure” (7) and “Extracellular structures” (6) were the smallest COG categories. KEGG pathway mapping was also carried out. The results showed that 24,651 unigenes were mapped to 128 predicted metabolic pathways (Figure 4; Table S4). The largest category was “Metabolism” including “Global map” (8,736), “Carbohydrate metabolism” (3,464), “Lipid metabolism” (2,955), “Amino acid metabolism” (1,390), “Biosynthesis of other secondary metabolites” (1,105), “Nucleotide metabolism” (1,033), “Energy metabolism” (936), “Metabolism of terpenoids and polyketidesgly” (818), “Glycan biosynthesis and metabolism” (619), “Metabolism of cofactors and vitamins” (586), and “Metabolism of other amino acids” (521) (Figure 4, A class; Table S4).

### Differential expression analysis of assembled *L. aurea* transcripts under MeJA treatment

**Figure 5:**
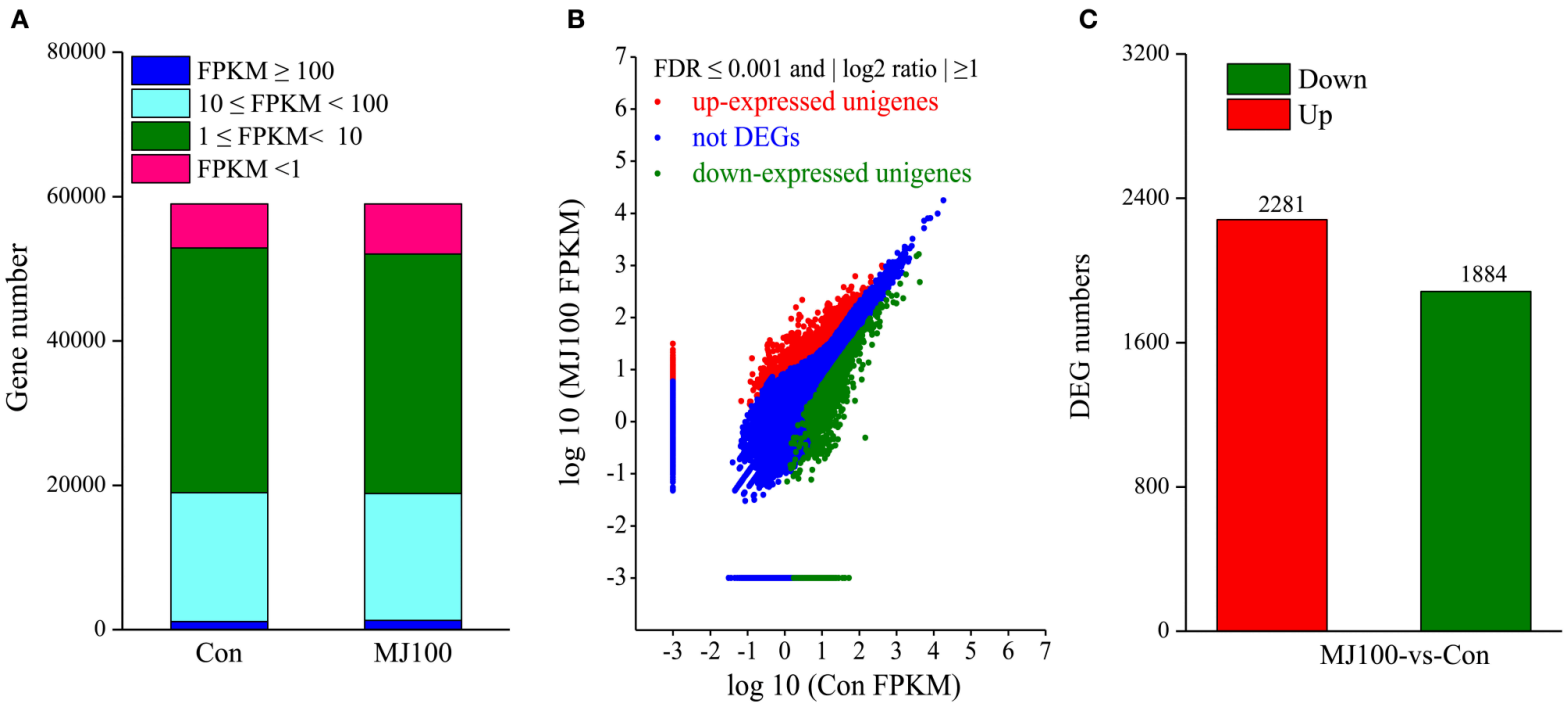
**Transcriptomes of ***L. aurea*** under MeJA treatment. (A)** Number of unigenes expressed in each sample. **(B)** Scatter-plot graphs of the differential gene expression patterns between Con and MJ100 libraries. DEGs were determined using a threshold of FDR ≤ 0.001 and |log2 Ratio| ≥ 1. Red spots represent up-regulated DEGs and green spots indicate down-regulated DEGs. Those shown in blue are unigenes that did not change significantly under MeJA treatment. **(C)** Number of differentially expressed genes (DEGs) showing up- (red) or down- (green) regulation between the samples.

To identify transcriptional responses of unigenes under MeJA treatment, reads from Con and MJ100 samples, were mapped to the obtained non-redundant unigenes. According to the FPKM values, the mappable reads were used to estimate the transcription levels. More than 97.0% of unigenes had FPKM values in the range of 1–100 (Figure 5A). Then the expression levels of unigenes in both samples were calculated. The unigenes which had at least a two-fold change with FDR ≤ 0.001 (Figure 5B) were screened and taken as differentially expressed genes (DEGs). Totally, we identified 4,165 DEGs between Con and MJ100 samples. Among them, 2,281 DEGs were found up-regulated and 1,884 down-regulated (Figure 5C).

### Functional classification of the DEGs

To further elaborate the functions of DEGs, we performed GO enrichment analysis, using Fisher's exact test with an FDR adjusted *P* ≤ 0.01 as the cutoff. Of the 4,165 DEGs, 1,806 were assigned GO annotations (Table S5). For example, In the BP category, “cellular process,” “metabolic process,” “single-organism process,” and “response to stimulus” were the top-four DEGs group (56.14%). In the CC category, the DEGs were annotated to “cell,” “cell part,” and “organelle” comprised the largest proportion (70.28%). Moreover, in the MF category, the genes which were associated with “catalytic activity” and “binding” took the biggest part (84.72%) of the DEGs (Table S6). Additionally, these DEGs were similarly enriched in the BP categories, such as “metabolic process,” “response to stimulus,” and “cellular process” (Table S6).

KEGG pathway enrichment analysis for DEGs was also performed. Of the 4,165 DEGs, 1,547 genes were assigned a KEGG ID and categorized into 121 pathways (Table S5). Of these, 29 pathways were significantly overrepresented under MeJA treatment, containing “Biosynthesis of secondary metabolites,” “Glycerophospholipid metabolism,” “Metabolic pathway,” “Plant hormone signal transduction,” and “Plant-pathogen interaction” (Table S7).

### Identification of MeJA-responsive genes expressed during MeJA treatment

**Figure 6:**
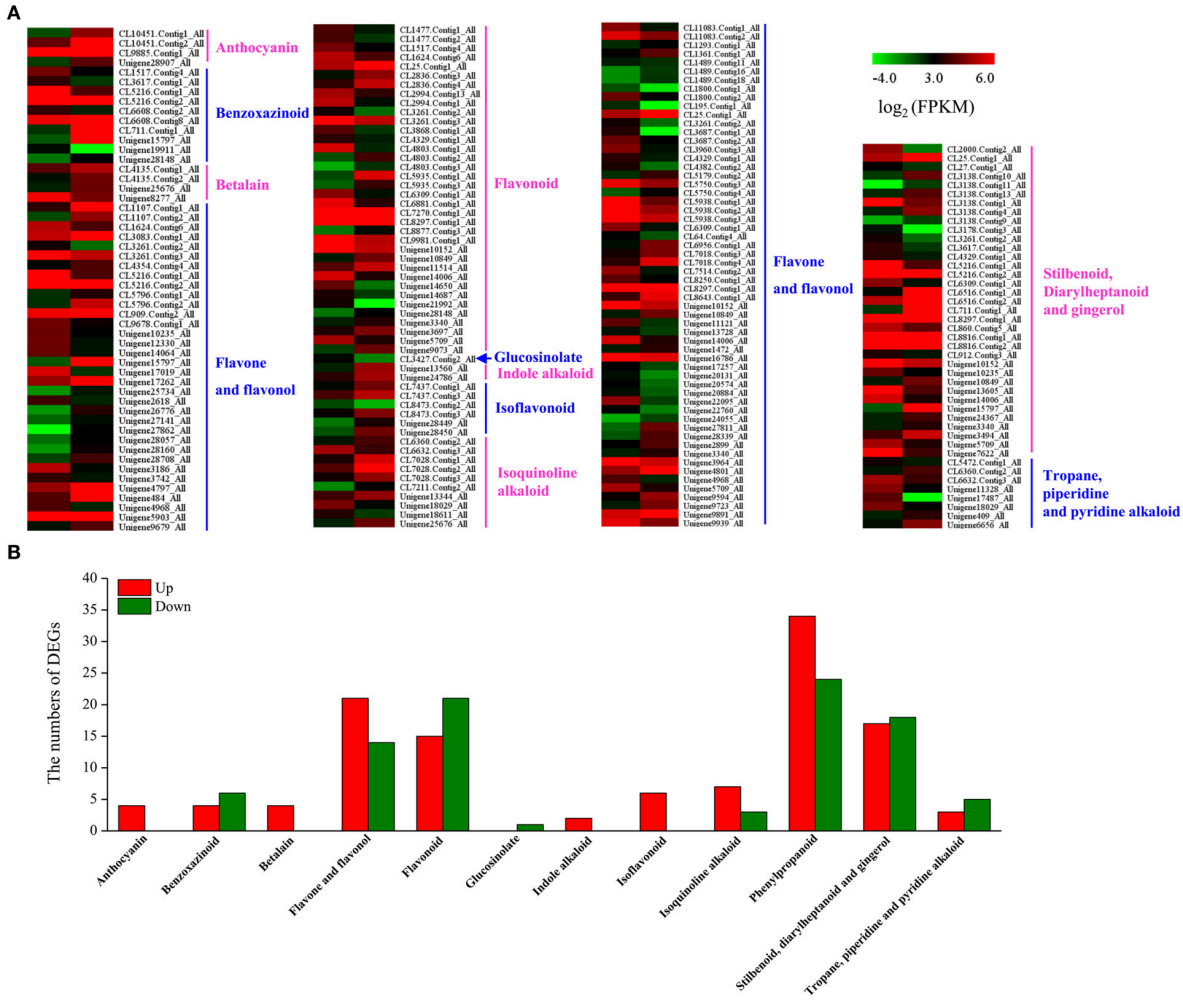
**Expression profiling of DEGs in secondary metabolism pathway. (A)** List of DEGs involved in different secondary metabolism pathway under MeJA treatment. **(B)** Distribution of DEGs involved in different secondary metabolism pathway under MeJA treatment.

**Figure 7:**
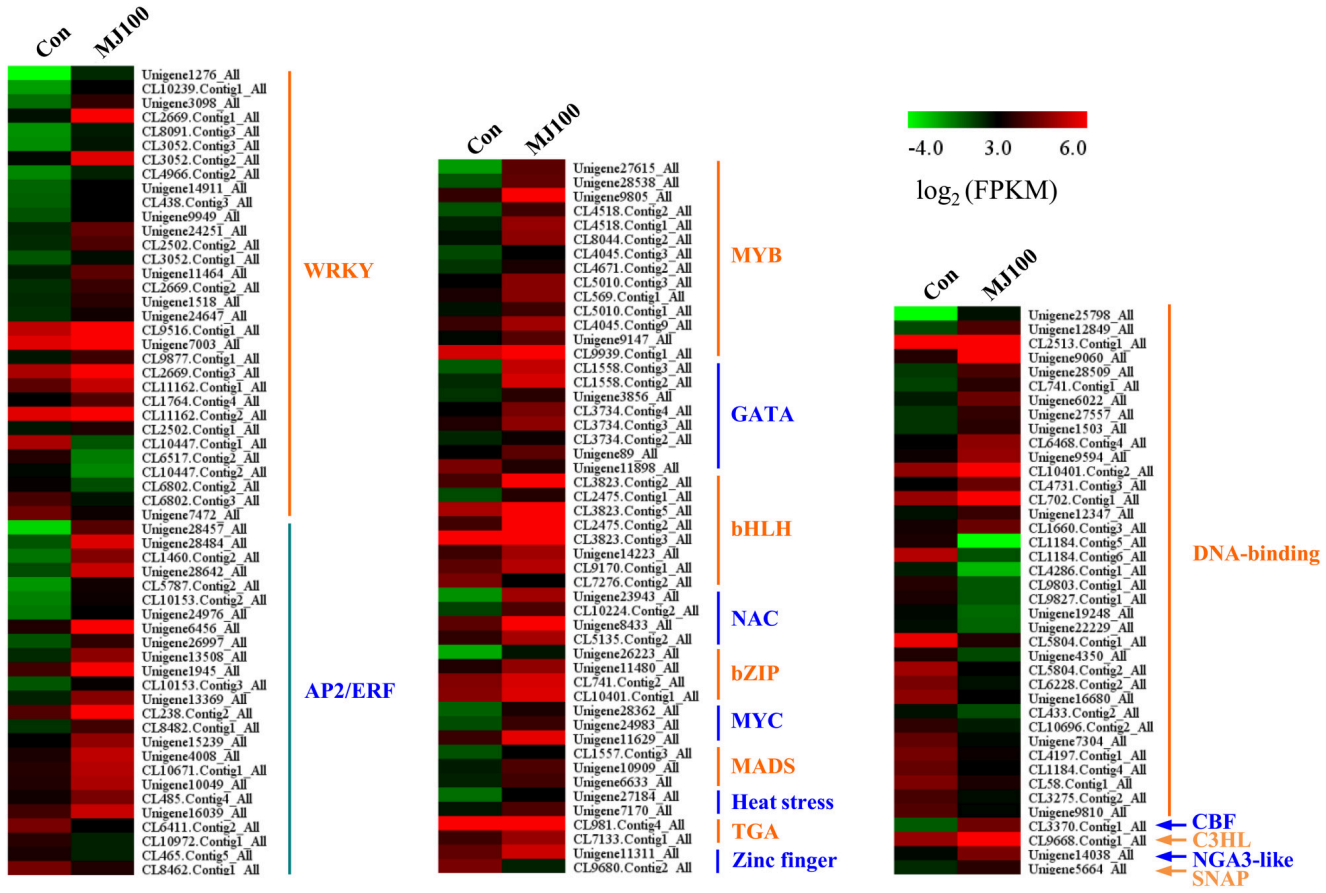
**Expression profiling of DEGs annotated as transcription factors**.

**Figure 8:**
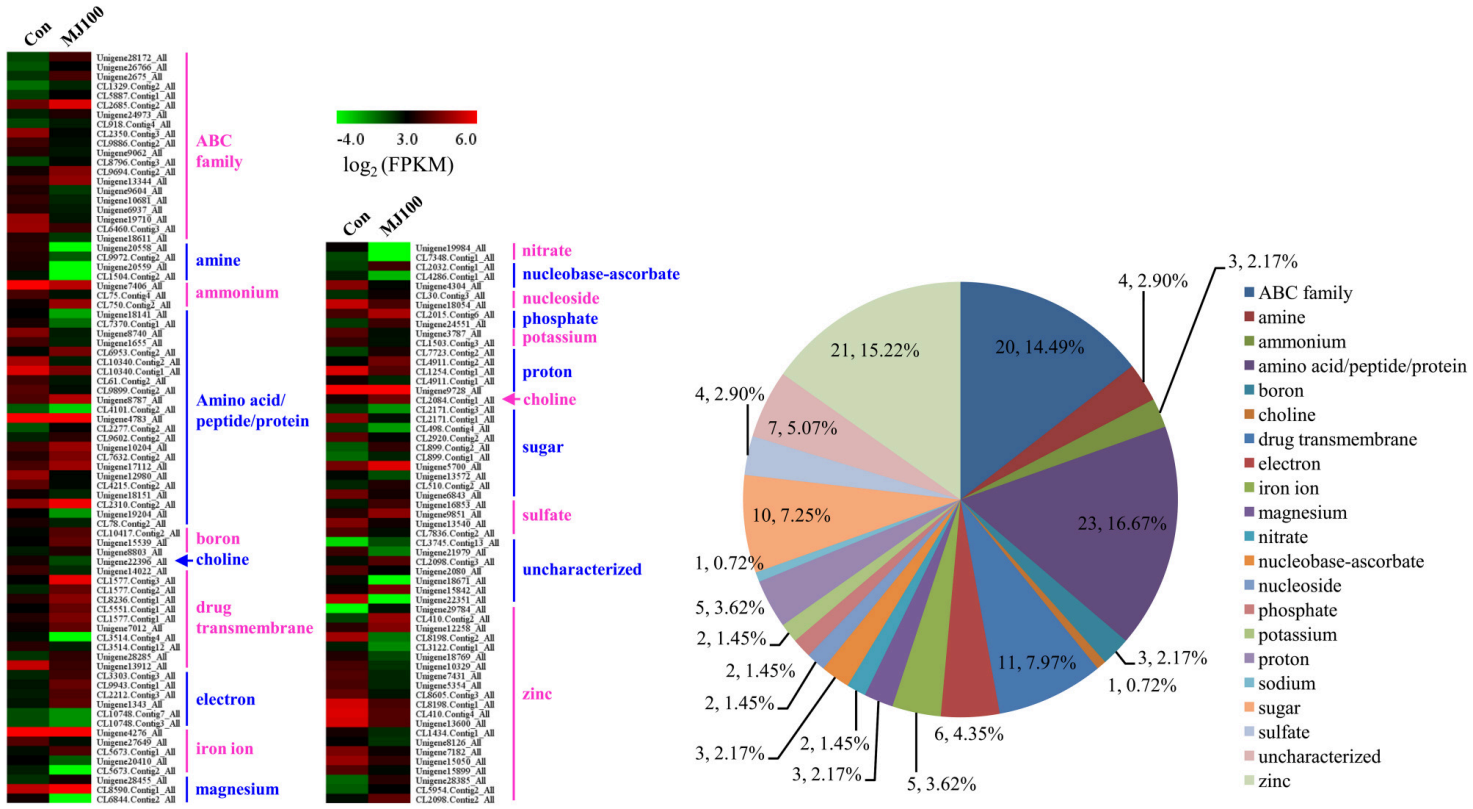
**Expression profiling of DEGs annotated as transporters**.

We analyzed 209 DEGs involving in 12 biosynthesis pathways of secondary metabolites (Figure 6). The results showed that the largest subcategory was phenylpropanoid biosynthesis (58), followed by flavonoid biosynthesis (36), flavone and flavonol biosynthesis (35), and stilbenoid, diarylheptanoid and gingerol biosynthesis (35). To identify MeJA-related genes in *L. aurea*, we used unigene sequences in BLAST searches of the public databases, and found 1,591 and 2,111 unigenes encoding transcription factors (TFs) and transporter proteins (TPs). Among them, 147 and 138 DEGs of TFs and TPs were detected respectively (Tables S5, S8). In total, the DEGs of TFs were divided into 17 subfamilies including WRKY, APETALA2/Ethylene-Response Factors (AP2/ERF), basic Helix-Loop-Helix (MYBs), bZIPs, and so on. Most of them were extensively up-regulated in response to MeJA treatment in *L. aurea* (Figure 7; Table 3). For example, 26 out of 32 WRKY TFs and all the 14 MYB TFs are up-regulated under MeJA treatment (Table 3). Meanwhile, numerous genes encoding transporters were also included in the sets of DEGs we detected (Table S8). The results showed that, under MeJA treatment, the expression level of 138 transporter genes varied (Figure 8). Among them, zinc transporter is the largest cluster family (21, 15.22%), and most of them were down-regulated under MeJA treatment. Notably, the expression level of genes encoding ATP-Binding Cassette (ABC) transporters (20, 14.49%), ammonium transporters (3, 2.17%), amino acid/peptide/protein transporters (23, 16.67%), drug transporters (11, 7.97%), electron transporters (6, 4.35%), iron ion transporters (5, 3.62%), magnesium transporters (3, 2.17%), nucleobase-ascorbate transporters (3, 2.17%), nucleoside transporters (2, 1.45%), proton transporters (5, 3.62%), sugar transporters (10, 7.25%), sulfate transporters (4, 2.90%), and uncharacterized transporters (7, 5.07%) changed under MeJA treatment (Figure 8).

**Table 3:** **Regulated transcription factors (TFs) under the MeJA treatment**.

**TF family**	**Number**	**Percentage (%)**	**Up**	**Down**
WRKY	32	21.77	26	6
AP2/ERF	25	17.01	21	4
MYB	14	9.52	14	0
GATA^*^	8	5.44	7	1
bHLH	8	5.44	7	1
NAC	4	2.72	4	0
bZIP	4	2.72	4	0
MYC	3	2.04	3	0
MADS	3	2.04	3	0
Heat stress	2	1.36	2	0
TGA	2	1.36	2	0
Zinc finger	2	1.36	2	0
CBF	1	0.68	1	0
C3HL^*^	1	0.68	1	0
NGA3-like	1	0.68	1	0
SNAP	1	0.68	1	0
DNA-binding TF activity protein	36	24.49	19	17
Total	147	100	117	30

* *Represents subgroup of zinc finger family*.

### Verification of RNA-Seq data by qRT-PCR

**Figure 9:**
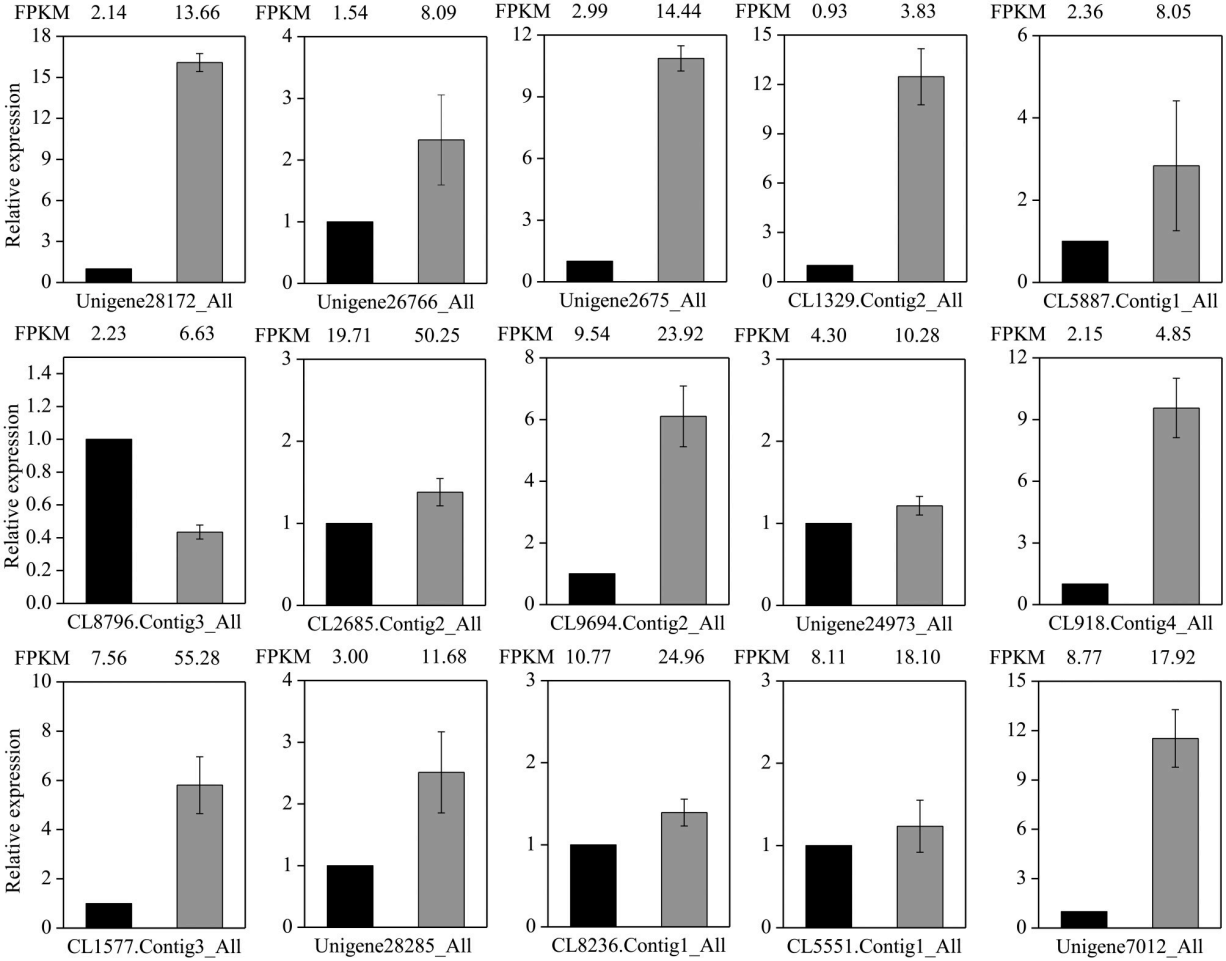
**Validation and comparative relative expression of 15 selected unigenes between the control and MeJA libraries in ***L. aurea*****. Differential expression between control (black column) and MeJA-treated sample (gray column) was compared. The normalized expression level (FPKM) of RNA-Seq is indicated on the x-axis. The relative expression levels were determined by quantitative real-time PCR using *Cyclophilin 1* as an internal reference; each PCR was repeated three times and the error bars represent standard deviation (SD).

To test the reliability of the RNA-Seq data, the expression level of 15 transporter genes were selected for qRT-PCR assays (Figure 9). These candidates included 10 ABC transporters and 5 drug transmembrane transporters (Table S1; Figure 8). The RNA-Seq data were compared with the transcript abundance patterns of the MeJA treatment and control. Our results showed that almost all of the expression comparisons of qRT-PCR assay were in fairly good match with the RNA-Seq data, even if the fold-change of some genes in their expression level detected by sequencing and qRT-PCR did not match perfectly. These data confirmed the reliability of the RNA-Seq results (Figure 9). Only one ABC transporter gene (CL8796.Contig3), which was analyzed by qRT-PCR, revealed significant difference in expression level comparing with the RNA-Seq data (Figure 9). In a word, the expression patterns of all unigenes consistent with the RNA-Seq data, indicating that our experimental results were reliable.

## Discussion

The RNA-Seq should lead to the transcriptional profiling analysis. By using Illumina HiSeq™ 2,000 sequencing, we got two libraries (Con and MJ100), producing 4.82 Gbyte and 4.65 Gbyte of clean data, respectively, which are larger than the previous 454 GS platform database (Wang et al., 2013). The transcriptome assembly was carried out by using Trinity software, which has good performing in none reference genome assembling (Grabherr et al., 2011). It showed that the short reads with average lengths of 244 and 273 bp of the two libraries, were collected into 181,881 and 156,621 contigs, respectively (Figure 1).

Sequencing profile is an effective tool to obtain functional genes (Wang et al., 2009). For example, inspection of the leaf epidermis enriched transcription database, an ABC transporter CrTPT2 that mediates the transporter of anticancer drug components in *Catharanthus roseus* was identified (Yu and Luca, 2013). Even more, combined the danshen (*Salvia miltiorrhiza*) EST database with the next-generation sequencing of mRNA from induced hairy root, Guo et al. (2013) identified CYP76AH1 which catalyzes turnover of multiradiene in tanshinones biosynthesis, leading to a successful heterologous production of ferruginol in yeast cell. In this study, combining with the previous EST database of *L aurea* (Wang et al., 2013), the entire transcriptome information obtained would be helpful for the future functional genomic research in *L. aurea*.

It has become clear that as an elicitor, MeJA is the main signal of secondary metabolite production across the plant kingdom, from angiosperms to gymnosperm (De Geyter et al., 2012). MeJA treatment triggers the majority of secondary metabolites biosynthesis (i.e., terpenoids, phenylpropanoids, and alkaloids) through an extensive transcriptional reprogramming (Zhao et al., 2005; Pauwels et al., 2009; De Geyter et al., 2012; Misra et al., 2014). Previously, we also showed that exogenous MeJA application accelerated the Amaryllidaceae alkaloids accumulation in *L. chinensis* seedlings (Mu et al., 2009). Here, by using trancriptome sequencing, we investigated the MeJA-responsive transcriptional changes, and identified 4,165 DEGs (including 2,281 up-regulated and 1,884 down-regulated) between Con and MJ100 samples in *L. aurea* (Figure 5C). They were categorized into 121 KEGG pathways (Table S5), and involved in 12 biosynthesis pathways of secondary metabolites (Figure 6). In general, Amaryllidaceae are regarded as derivatives of the common precursor 4′-*O*-methylnorbelladine via intramolecular oxidative phenol-coupling (Eichhorn et al., 1998; Park, 2014), which belongs to the isoquinoline alkaloid biosynthesis pathway. Recently, a norbelladine 4′-*O*-methyltrasferase (*Np*N4OMT) gene involved in the biosynthesis of galanthamine in *Narcissus* ap. *aff. pseudonarcissus* has been characterized (Kilgore et al., 2014). When aligned against transcriptome database, the potential homologous gene of *Np*N4OMT in *L. aurea* was also found, and its expression level showed more than two times higher under MeJA treatment, when compared with the control sample (Table S7). It suggested that DEGs database reserving a large number of genetic information which would be helpful for characterizing the key enzymes associated with the Amaryllidaceae alkaloids biosynthesis.

The expression of plant secondary pathway is under the tight control of a large number of TFs at different levels (Yang et al., 2012). For example, TFs involved in Jasmonates (JAs) signaling cascades usually regulate the transcription of multiple genes in a biosynthesis pathway, so as to improve the production of secondary metabolites (Zhou and Memelink, 2016). Several types of TFs shown as regulators of secondary metabolite biosynthesis in plants have been identified, belonging to the families AP2/ERF, bHLH, MYB, and WRKY (Naoumkina et al., 2008; Shoji et al., 2010; Todd et al., 2010; Yang et al., 2012; Yu et al., 2012; Misra et al., 2014). In this study, at least 1,591 unigenes encoding transcription factors (TFs) were found. Among them, 147 DEGs of TFs were simultaneously detected (Tables S5, S8). They were divided into 17 subfamilies, and most of them were extensively up-regulated in response to MeJA treatment (Figure 7; Table 3), suggesting their possible involvement in the regulation of secondary metabolite biosynthesis in *L. aurea*. The WRKY TF family is unique to plants, and is characterized by a conserved WRKY domain which specifically binds to the W-box sequence (Rushton et al., 2010; Zhou and Memelink, 2016). Previous reports indicated that many WRKY TFs may be regulated by wound and JA signal, and are reported as regulators of genes involved in various secondary metabolite pathways (Chen et al., 2012; Phukan et al., 2016). As shown in this study, among the DEGs, WRKY cluster is the largest family and most of them were up-regulated under MeJA treatment (Table 3; Figure 7). This result suggested the potentially important role of WRKY TFs in the regulation of gene expression in *L. aurea*. In addition, the bHLH TF MYC2 has been reported as a master regulator in the JAs signaling network (Kazan and Manners, 2013). Transcription levels of the key enzymes of the primary and secondary metabolism were also regulated by MYC2 (Dombrecht et al., 2007). In this study, we also noticed that most of the bHLH TF genes (including 3 MYC genes), were up-regulated under the MeJA treatment in *L. aurea* (Figure 7). The expression changes of these transcription factors may reveal their key functions.

The verity of secondary metabolites has a great deal of differences among the species; the specific compounds are often restricted in a few species, or even within a few varieties within a species (Smetanska, 2008). Moreover, the biosynthesis and storage of these compounds are usually tissue- and developmental stage-specific. In plants, the secondary metabolites always have to be stored in the special tissues or subcellular compartment which is distinct from the biosynthesis part. The mechanism of the biosynthesis and accumulation of plant secondary metabolites in such appropriate pattern attracted more attention (Yazaki et al., 2008; Nour-Eldin and Halkier, 2013). In most cases, plant secondary metabolites are transported intercellularly, intracellularly, and in an intratissue fashion by specific transporters (Yazaki, 2005; Yazaki et al., 2008; Nour-Eldin and Halkier, 2013). Meanwhile, the membrane transporter is comparatively specific and highly controlled for each secondary metabolite (Yazaki, 2005; Nour-Eldin and Halkier, 2013; Lv et al., 2016). ABC transporter family, based on hydrolysis of ATP, is proved to take a main part of transporting the secondary metabolites (Yazaki, 2005, 2006). Since many plant secondary metabolites are medicinally used, the ABC transporters are associated with the drug resistant (Fletcher et al., 2010). In addition, the ABC transporters that presumably take part in secondary metabolite transport were also characterized among the MeJA up-regulated transcripts. For example, the ABCG transporter unigenes related to artemisinin yield in *Artemisia annua*, which are responsive to methyl jasmonate treatment, have been identified (Zhang et al., 2012). In *Panax ginseng*, a novel PDR-type ABC transporter gene *PgPDR3* induced by MeJA was also characterized (Zhang et al., 2013). It has been suggested that ABC transporters are often associated with the special compound transport, including alkaloids, terpenoids, and polyphenols, etc. (Sakai et al., 2002; Yazaki, 2005, 2006; Yazaki et al., 2008; Shoji, 2014; Lv et al., 2016). Among the DEGs of *L. aurea* treated with MeJA, a large proportion of ABC transporters were identified (Figure 8; Table S8). Thus, the results will be helpful to identify the potential ABC transporters for translocation of secondary metabolites in *L. aurea*.

In this study, a large-scale unigene investigation of *L. aurea* under MeJA treatment was performed by Illumina sequencing. In total, we found 4,175 DEGs, and many transcripts were encoded by putative genes including transporter proteins, transcription factors and enzymes involved in secondary metabolism pathway. The data we obtained provided comprehensive information on gene discovery, transcriptome profiling, and transcriptional regulation of *L. aurea*. Additionally, our findings highlight the significance of JA signaling and Amaryllidaceae alkaloids synthesis in *L. aurea*, and provide a foundation for subsequent genomic research in the future.

## Author contributions

ReW designed the project, performed the experiments and wrote the manuscript. RoW, SX, and NW analyzed the data and wrote the manuscript. BX and YJ provided the plants and revised the manuscript. All authors read and approved the final manuscript.

### Conflict of interest statement

The authors declare that the research was conducted in the absence of any commercial or financial relationships that could be construed as a potential conflict of interest. The reviewers ZM, JZ and handling Editor declared their shared affiliation, and the handling Editor states that the process nevertheless met the standards of a fair and objective review.

## Abbreviations

DEG: differentially expressed gene
FDR: false discovery rate
RNA-Seq: high throughput sequencing of cDNA libraries
cDNA: complementary DNA synthesized from RNA
qRT-PCR: quantitative real-time polymerase chain reaction
GO: gene ontology
KEGG: Kyoto encyclopedia of genes and genomes
Nr: non-redundant database
COG: cluster of orthologous groups databases
CYP1: cyclophilin 1
NCBI: National Center for Biotechnology Information
MS: Murashige and Skoog
FPKM: Fragments per kilobase of exon per million fragments mapped reads
ABC: ATP-Binding Cassette.
