# Supplementary material for: Transcriptome Analysis of Secondary Metabolism Pathway, Transcription Factors, and Transporters in Response to Methyl Jasmonate in Lycoris aurea
Source: Front Plant Sci. 2017 Jan 5;7:1971. doi: 10.3389/fpls.2016.01971 (PMC5217099; doi:10.3389/fpls.2016.01971)
Supplement: Supplementary file 1 [file Table1.doc]

**Table S1** qRT-PCR primers used in this study

| Primer name | Gene annotation | Primer sequence (5’-3’) |
| --- | --- | --- |
| Unigene28172_All F | ABC transporters | CCCCTGAGTGTCGGTTTGAT |
| Unigene28172_All R |  | TTACATGGCATTCCACACTTTCTC |
| Unigene26766_All F |  | CGCCAATGCACTAGTTTTCG |
| Unigene26766_All R |  | CGGCATAGAGAAAAGGATCGA |
| Unigene2675_All F |  | ACCTAGTGAAGCTCCAGAGGTTGT |
| Unigene2675_All R |  | CCTACAGCCGGCCAACAT |
| CL1329.Contig2_All F |  | TGACGCAGCGCTGTCTAGAC |
| CL1329.Contig2_All R |  | CGCCCGAAGCTCCTGTT |
| CL5887.Contig1_All F |  | GTCCAGCAAGGCCATCAAAT |
| CL5887.Contig1_All R |  | GGCCTCGGCAGCAAGTTT |
| CL8796.Contig3_All F |  | GCTAATTGCACTAAACCCGGTAA |
| CL8796.Contig3_All R |  | CAATCTCGTGTTTTCGGGTAAGA |
| CL2685.Contig2_All F |  | GACCGTGCACTCTGCGAAT |
| CL2685.Contig2_All R |  | TGCCACCGATGTGATCCTT |
| CL9694.Contig2_All F |  | ATGCATGGCTTGCCTATGG |
| CL9694.Contig2_All R |  | CTGCGTTGTAAGGAGGATTCCT |
| Unigene24973_All F |  | AAAAGAGGCTCCTCAACAGAACTC |
| Unigene24973_All R |  | TTATGCCGCGGTTTTTGG |
| CL918.Contig4_All F |  | TCATGATCGGGTCCCTGATC |
| CL918.Contig4_All R |  | GAGCTGCAGAGTGTGCATTCTAG |
| CL1577.Contig3_All F | Drug transmembrane transporters | ACGAAGGCTTGCCAACCA |
| CL1577.Contig3_All R |  | CGGTGTCCAGCCAGTCCTAT |
| Unigene28285_All F |  | ATAGGCCAAGAACCTTTAATCTCACT |
| Unigene28285_All R |  | AAGGCCAGGGATCATCCATAG |
| CL8236.Contig1_All F |  | CGACAGCGTTCTTTGCTTTCT |
| CL8236.Contig1_All R |  | CGCGCGTGTCAAATGAGA |
| CL5551.Contig1_All F |  | CCGAGGGCAACCGAGAA |
| CL5551.Contig1_All R |  | ACCGCCAGCAGCTTCCT |
| Unigene7012_All F |  | ATCCCAGTGATCACATTCTTTTTG |
| Unigene7012_All R |  | AGACCCTTTTCAGGTTGACAGTTT |
