# Supplementary material for: Transcriptome Analysis of Secondary Metabolism Pathway, Transcription Factors, and Transporters in Response to Methyl Jasmonate in Lycoris aurea
Source: Front Plant Sci. 2017 Jan 5;7:1971. doi: 10.3389/fpls.2016.01971 (PMC5217099; doi:10.3389/fpls.2016.01971)
Supplement: Supplementary file 5 [file Table5.DOC]

**Table S5** Numbers of annotated genes and expressed features

| All/DEG | Unigenes | GO | KEGG | No. of expressed features | |
| --- | --- | --- | --- | --- | --- |
|  |  |  |  | TF | TP |
| All | 59643 | 31157 | 24651 | 1591 | 2111 |
| DEG | 4165 | 1806 | 1547 | 147 | 138 |

DEG, differentially expressed gene; TF, Transcription Factor; TP, Transporter Protein.
